# The Intersection of Non-Coding RNAs Contributes to Forest Trees’ Response to Abiotic Stress

**DOI:** 10.3390/ijms23126365

**Published:** 2022-06-07

**Authors:** Dandan Xiao, Min Chen, Xiaoqian Yang, Hai Bao, Yuzhang Yang, Yanwei Wang

**Affiliations:** 1 National Engineering Research Center of Tree Breeding and Ecological Restoration, Key Laboratory of Genetics and Breeding in Forest Trees and Ornamental Plants, Ministry of Education, The Tree and Ornamental Plant Breeding and Biotechnology Laboratory of National Forestry and Grassland Administration, College of Biological Sciences and Biotechnology, Beijing Forestry University, Beijing 100083, China; xiaodd003@bjfu.edu.cn (D.X.); m-chen15@mails.tsinghua.edu.cn (M.C.); xiaoqianyang51@163.com (X.Y.); haibaobiology@163.com (H.B.); yangyuzhang@bjfu.edu.cn (Y.Y.); 2 School of Life Sciences, Tsinghua University, Beijing 100084, China

**Keywords:** abiotic stress, miRNA, lncRNA, circRNA, intersection, forest trees

## Abstract

Non-coding RNAs (ncRNAs) play essential roles in plants by modulating the expression of genes at the transcriptional or post-transcriptional level. In recent years, ncRNAs have been recognized as crucial regulators for growth and development in forest trees, and ncRNAs that respond to various abiotic stresses are now under intense study. In this review, we summarized recent advances in the understanding of abiotic stress-responsive microRNAs (miRNAs), long non-coding RNAs (lncRNAs), and circular RNAs (circRNAs) in forest trees. Furthermore, we analyzed the intersection of miRNAs, and epigenetic modified ncRNAs of forest trees in response to abiotic stress. In particular, the abiotic stress-related lncRNA/circRNA–miRNA–mRNA regulatory network of forest trees was explored.

## 1. Introduction

Non-coding RNAs (ncRNAs), accounting for a large and significant proportion of eukaryotic transcriptomes, have minimal or no protein-coding capacity but are functional, and can be classified into three types: small RNAs with 18–30 nucleotides (nt), medium-sized ncRNAs with 31–200 nt, and long ncRNAs (lncRNAs) (>200 nt) [1]. Plant primary miRNAs (pri-miRNAs) are transcribed by RNA polymerase II (Pol II) and processed by dicer-like1 (DCL1) to generate precursor miRNAs (pre-miRNAs), and then 21-nt mature miRNAs [2]. Intriguingly, plants might have another miRNA processing pathway, in which 24-nt miRNAs are generated by DCL3. Such miRNAs can be distinguished from small interfering RNAs (siRNAs) by their independence from RNA-dependent RNA polymerase 2 (RDR2) [3,4]. Plant miRNAs are loaded into Argonaute (AGO) proteins to form the RNA-induced silencing complex (RISC), which regulates gene expression at the transcriptional or post-transcriptional level and is involved in multiple developmental signaling pathways [5]. Investigations have confirmed that *REDUCTION IN BLEACHED VEIN AREA* (*RBV*), encoding a nuclear WD40 domain protein, promotes miRNA biogenesis at the *MIR* gene transcription and AGO1 loading steps, and may also enhance pri-miRNA processing in *Arabidopsis* [6]. miRNAs play crucial roles in tissue-specific and environmentally-induced gene repression. LncRNAs can be classified as long intergenic non-coding RNAs (lincRNAs), long non-coding natural antisense transcripts (lncNATs), long intronic non-coding RNAs, and overlapping lncRNAs, based on their genomic positions relative to protein-coding genes [7]. LncRNAs have tissue-specific levels of expression, either promoter- or enhancer-associated, and play important roles in the regulation of transcription and translation of coding RNA in the vicinity, including dosage compensation, genomic imprinting, maintenance of genome integrity, cell cycle control, development, and differentiation [8,9]. Unlike linear RNAs, circular RNAs (circRNAs), a type of new regulatory RNA with diverse biological functions, mainly arise from exons (exonic circRNA) or introns (intronic circRNAs) and are differentially generated by back splicing or lariat introns [10]. NcRNAs have emerged as essential regulators in response to abiotic stress in plants [11]. Thus, it is necessary to discuss and summarize the diverse functions of ncRNAs of plants in response to abiotic stress.

Thus far, the general and specific roles of ncRNAs in response to abiotic stress have been widely studied in *Arabidopsis* and crops, based on their genomic integrity and readily molecularly manipulated characteristics. A review of plant survival in a variable nutrient environment proposed a signaling model participated in by miR399 and miR827 in the systemic phosphorus (Pi) starvation response in plants by regulating the PO_4_^−^ transporter, demonstrating the crucial roles of both miR399 and miR827 in nutrient deficiency [12]. miR172 positively regulates salt tolerance in both rice and wheat, suggesting general conserved characteristics in crop species. Intriguingly, miR172a and miR172b, but not miR172c or miR172d, were found to be involved in the salt stress response, showing the differential functions of the miR172 family. Further investigation uncovered that the miR172/IDS1 signaling module confers salt tolerance through maintaining reactive oxygen species (ROS) homeostasis in cereal crops [13]. Interestingly, studies have revealed that an array of miRNAs could be general regulators in the defense against stressful environments, such as miR408. miR408 is a highly conserved miRNA among plant species and responds to the availability of copper, and its target genes encode copper-containing proteins [14]. Furthermore, miR408 was found to be involved in various abiotic stress processes, including salinity, cold, oxidative stress, nutrient deficiency, and drought [14,15,16]. Although large-scale investigations have uncovered the essential functions of ncRNAs in annual plants in response to abiotic stress, related studies on how ncRNAs regulate forest trees under environmental stress are lacking. Here, we focused on the frontiers of abiotic-stress-responsive ncRNAs, and especially the regulatory network among ncRNAs in forest trees.

## 2. miRNAs Involved in the Response to Abiotic Stress

### 2.1. Drought Stress-Responsive miRNAs

Drought stress is one of the most critical environmental factors affecting plant growth and development. Recently, miRNAs have emerged as game-changers in regulating forest trees’ response to drought stress. Various miRNAs and their potential target genes are differentially expressed in *Populus* (*P*.) *trichocarpa* in response to drought stress, such as miR167, miR168, miR396, and miR164, pointing to their essential roles in the drought response [17,18]. Using high-throughput sequencing and microarray analysis, 104 and 27 miRNAs were found to be upregulated and downregulated, respectively, by drought stress in *P*. *euphratica* [19]. In a similar study with *P*. *tomentosa* and drought stress treatment, we found 17 conserved miRNA families and nine novel miRNAs that were significantly changed [20]. In *P. trichocarpa*, 91 phasiRNAs derived from 20 PHAS loci were identified, and about half of them were responsive to drought stress, including six PHAS initiated by specific miRNAs, such as miR6445 and miR6427, in *Populus* [21]. Recently, miRNAs were also found to modulate the response of diploids and autotetraploids of *Paulownia tomentosa* to drought through sRNA and degradome sequencing analysis [22,23]. These investigations were mostly based on high-throughput sequencing and microarray, and confirmed the universality and diversity of miRNAs in the modulation of the response of forest trees to drought stress. Furthermore, a few miRNAs and their targets have been verified in transgenic *Populus* in response to drought stress, including miR169o and its target subunit A of Nuclear Factor Y (*PtNF*-*YA6*), miR172d and its target GT-2-like 1 (GTL1) trihelix transcription factor (*PuGTL1*), miR472a and its target F-box 1 (*FB1*), and miR6445 and its target *NAC* (NAM, ATAF, and CUC) transcription factor [24,25,26]. However, detailed functional analyses and understanding of the regulatory mechanisms of miRNAs in forest trees are yet to be reached, in contrast with the annual model plant *Arabidopsis* and rice.

### 2.2. Cold and Heat Stress-Responsive miRNAs

Cold stress, including chilling (>0 °C) and freezing (<0 °C), tremendously impacts the growth, development, and distribution of forest trees. miRNAs have been widely found to be responsive to cold stress in forest trees [27]. In *P. trichocarpa*, miR169, miR172, miR393, and miR395 showed different expression patterns under cold stress in microarray analysis [28]. High-throughput sequencing identified 144 conserved miRNAs belonging to 33 miRNA families, and 29 novel miRNAs (as well as their corresponding miRNA*s) belonging to 23 miRNA families, as well as 30 miRNAs that were differentially expressed in response to cold stress in *P*. *tomentosa*. Among them, 19 conserved and 2 novel miRNAs and their corresponding miRNA*s expressions were validated by qRT-PCR [29]. In *P. suaveolens*, time course expression analysis of transgenic plants overexpressing miR475 uncovered that Psu-miR475b promoter mediates the transcriptions of *Psu-miR475b*, and its targets might be involved in a crosstalk between cold stress and other stress signaling processes, indicating multiple functions of the same miRNA in different stresses [30]. The application of degradome sequencing confirmed 80 genes to be the targets of 51 unique miRNAs, including three downregulated miRNAs (pto-miR156k, pto-miR169i-m, and pto-miR394a-5p/b-5p) and two upregulated miRNAs (pto-miR167a-d and pto-miR167f/g); the results might be a good basis for further research on miRNA-mediated regulatory mechanisms and molecular improvement of resistance to cold stress in poplar [31]. Expression profiling of small RNAs found that a series of miRNAs were responsive to chilling treatment, including miR319, miR156, miR172, miR160, and miR1444, providing evidence of miRNAs involved in the regulation of the dormancy-active growth transition of trees [32,33,34]. These investigations showed that miRNAs play an important role in the regulation of the cold stress response in *Populus.*

Heat stress (non-lethal high temperatures of 37–42 °C) profoundly affects plant growth and development [35]. More emerging miRNAs have been found to play important roles in plant responses to heat stress [36]. In *P*. *tomentosa*, 52 miRNAs from 15 families were found to be responsive to heat stress and most of them were downregulated. Intriguingly, miR167c-d, miR168a-b, miR395a-j, and miR482 showed dynamic changes under a time-course heat-stress treatment [37]. miRNAs such as miR171l-n, miR1445, and miR1446a in *P. trichocarpa,* analyzed by RNA gel blots with end-labeled antisense oligonucleotides, were also heat-stress responsive [28]. Transgenic hybrid poplar overexpressing growth-regulating factor 15 (*GRF15*) and lacking the miR396a complementary sites exhibited enhanced heat tolerance and photosynthetic efficiency compared with wild-type plants [35]. In addition, miR398 and its target encoding copper/zinc superoxide dismutases (*CSDs*) were also found to be important in the heat stress responses of *P*. *tomentosa* [36].

### 2.3. Salt Stress-Responsive miRNAs

Considering the impact of increasing salt stress on the distribution and growth of plants, miRNAs involved in the salt stress response have been intensively investigated in diverse plants [38]. In *P. euphratica*, a great number of new miRNAs have been discovered, and both known and novel miRNAs were found to functionally cleave their target mRNAs under short term (1 d) and long term (8 d) salt stress through the combination of the small RNAome, degradome, and transcriptome, indicating that expression of miRNAs and targets were correspondingly induced or suppressed by salt stress [39]. Other research revealed that miRNAs responsive to salt stress in the roots were more sensitive than those in the leaves, and were distributed widely in diverse tissues and differentially expressed under different salt conditions in *P. euphratica* [40]. To identify miRNAs in *P*. *tomentosa* treated or not with salt (200 mM NaCl for 10 h), high-throughput sequencing also detected 21 conserved miRNAs and 7 non-conserved miRNAs that were differentially expressed, providing new insights into salt-responsive miRNAs in *Populus* [41]. In *P. cathayana* (salt-sensitive type) and *Salix matsudana* (salt-tolerant type), microarray analysis found 161 and 32 responsive miRNAs, respectively, under salt stress. Interestingly, this investigation further revealed the different change patterns of the same miRNAs in the two species, such as ptc-miR474c and ptc-miR398b, implying that miRNAs might have varying responses to salination conditions in different species [42]. In transgenic *Populus*, *miR390*/*TAS3*/*ARFs* were confirmed to modulate lateral root growth under salt stress via the auxin pathway [43]. The diploids and allotriploids of *P*. *cathayana* were treated with 70 mM NaCl solution for 30 days, while 22 differentially expressed miRNAs were significantly correlated with salt-stress response genes, which indicated that miRNAs were involved in the poplar response to salt stress [44]. Taken together, an increasing number of studies have revealed that miRNAs play key roles in the response to salt stress in forest trees, enhancing our understanding of the molecular mechanisms of salt resistance and helping to elucidate new miRNA members involved in stress response pathways in *Populus*.

### 2.4. Nutrition Stress-Responsive miRNAs

There is ample evidence that miRNAs play important roles in the processes of plant adaption to nutritional stress through signaling and regulating nutrient transport and utilization [45]. Pi and sulfur deficiency investigations have suggested the existence and conservation of miR395 and miR399 and their target genes among a number of plant species, implying the evolutionary importance of miRNA-mediated regulation of nutrient stress responses [46]. Under low nitrogen (N) conditions, 95 miRNAs belonging to 21 conserved families were differentially expressed in *P. tomentosa*, including pto-miR319, pto-miR393, pto-miR395, and pto-miR396, which were induced, while the rest were suppressed. This indicated the miRNAs are responsive to N stress in *Populus* [47]. The abundance of 65 known and 3 novel miRNAs changed dramatically under Pi deficiency in *P*. *tomentosa*; miR167, miR394, miR171, and miR857 were responsive to both low N and low Pi environments, demonstrating that the same miRNA could mediate the response of different nutrition stresses [47]. Moreover, this investigation also showed that the decreased abundance of almost 50% of the known and novel miRNAs under Pi deficiency could be restored through sufficient Pi addition to the environment, further providing an explanation for symptom recovery in plants under adverse nutrition stress with the appropriate environmental improvement [48]. Integrated analysis of mRNA-Seq, miRNA-Seq, and degradome-Seq indicated that miRNA showed tissue-specific characters and allowed deciphering miRNA functions and establishing a framework for exploring Pi signaling networks regulated by miRNAs in *Betula luminifera* [49]. Taken together, in tree plantations, master regulator miRNAs that can improve nutrient utilization efficiency show diverse and important roles in the ecology of forest trees, and should receive more attention in the future.

### 2.5. Oxidative and Hypoxic Stress-Responsive miRNAs

Oxygen is an indispensable substrate for many biochemical reactions in plants, including energy metabolism (respiration) [50]. Stress conditions such as drought, cold, salinity, heat, and heavy metals result in excess ROS accumulation in plants, which may disrupt the balance between ROS production and scavenging under normal conditions [51]. The ectopic expression of copper/zinc superoxide dismutase (*CSD1* and *CSD2*), targeted by miR398, mediates the responses of grapevines to copper (Cu) stress with lower levels of ROS and higher levels of superoxide dismutase (SOD) accumulation in the transgenic lines [52]. Hypoxic stress, mainly caused by flooding events, significantly reduces the efficiency of cellular ATP production, which has diverse ramifications for cellular metabolism and developmental processes in plants [53]. In *P. tomentosa*, significant changes in the expression of seven conserved miRNA families and five novel miRNAs were observed in response to flooding stress, providing evidence of miRNAs mediating the hypoxic stress response in forest trees. Additionally, both miRNAs and miRNA*s were found to participate in the regulation of tree responses to water stress [20].

### 2.6. UV-B Stress-Responsive miRNAs

Trees are sessile organisms and are inevitably exposed to intense sunlight outdoors, including ultraviolet radiation (UV, 280–400 nm), especially high-energy, short-wave length UV-B radiation (280–315 nm), which is a component of sunlight [54]. Plants sense natural UV-B radiation and respond rapidly to high levels of UV-B radiation, and regulation mediated by miRNAs is crucial [55]. A series of UV-B stress-responsive miRNAs (13 upregulated and 11 downregulated) was identified in *P*. *tremula* through miRNA filter array [56]. Northern blotting validated that the UV-B regulated miRNAs, including miR169, miR395, and miR472, were downregulated and miR168, miR398, and miR408 were upregulated under UV-B stress [56]. Furthermore, the regulatory network of miRNAs in response to UV-B was investigated in grapes. High-fluence UV-B induced miR168 and miR530, which target AGO1 and a Plus-3 domain mRNA, respectively, while suppressed miR403 targeting AGO2, thereby orchestrating post-transcriptional gene silencing activities by different AGOs [57]. miR395 and miR399 were positively responsive to UV-B light during grapevine berry development [57].

### 2.7. Intersection of Abiotic Stress-Responsive miRNAs

**Figure 1:**
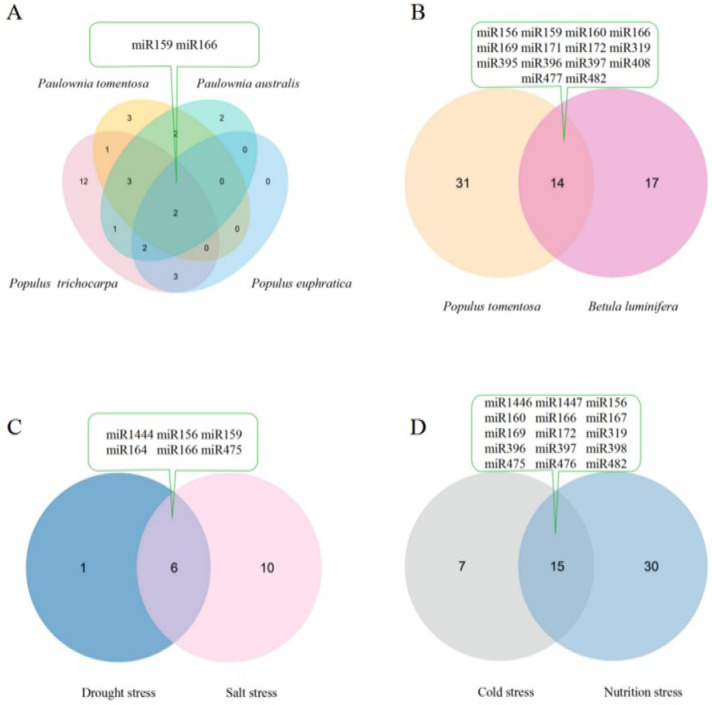
Intersection of abiotic stress responsive miRNAs verified by degradome sequencing in different forest tree species based on previous studies [17,19,21,23,31,39,48,49,58,59]. (**A**) Drought stress-responsive miRNAs in *Populus trichocarpa*, *Paulownia tomentosa*, *Paulownia australis*, and *Populus euphratica*; (**B**) Nutrition stress-responsive miRNAs in *Populus tomentosa* and *Betula luminifera*; (**C**) Drought and salt stress-responsive miRNAs in *Populus euphratica*; (**D**) Cold and nutrition stress-responsive miRNAs in *Populus tomentosa*. Note: the miRNAs in the green text box are the common content of intersection.

To comprehensively understand the role of miRNAs in forest trees in response to abiotic stress, we summarized and compared the related miRNAs and their target genes which had been verified by degradome sequencing from previously published studies (Supplementary Table S1) [17,19,21,23,31,39,48,49,58,59]. From the comparison, there were two drought-responsive miRNAs, miR159 and miR166, found in *P. trichocarpa*, *Paulownia tomentosa*, *Paulownia australis*, and *P. euphratica* (Figure 1A) [17,19,21,23,58]. miR159 mediates the response to drought stress in *P. trichocarpa*, *Paulownia tomentosa*, *Paulownia australis*, and *P. euphratica*, and the response to nutrition stress in *P*. *tomentosa* and *Betula luminifera*; miR166 mediates the response to cold and nutrition stress in *P*. *tomentosa*, drought, and salt stress in *P. euphratica*, drought stress in *P. trichocarpa*, *Paulownia tomentosa*, and *Paulownia australis*, and nutrition stress in *Betula luminifera* (Figure 1). Usually, miRNAs play important roles in plant responses to environmental stress by recognizing their target mRNAs by base pairing, resulting in cleavage or translational attenuation. miR159 might affect the expression of its target *GAMYB* in order to enhance drought resistance, peroxidase 21 precursor family protein-coding gene and *GAMYB* to enhance nutrition stress resistance, and *MYB65* and *MYB33* to enhance salt tolerance. Additionally, miR166 might affect the expression of its targets encoding homeobox-leucine zipper family protein/lipid-binding START domain-containing protein (ATHB-15) for the purpose of drought, nutrition, and salt resistance. There were 14 miRNAs related to nutrition stress in both *P*. *tomentosa* and *Betula luminifera* (Figure 1B) [48,49,59]. These results indicated the general conserved characters of miRNAs responsive to abiotic stress among different tree species. There were six miRNAs, including miR156 targeting the *SPL* family and miR164 targeting *NAC1*, which take part in coping with drought and salt stress in *P*. *euphratica* (Figure 1C) [19,39]. Additionally, 15 miRNAs are cold and nutrition stress responsive in *P*. *tomentosa* (Figure 1D) [31,48,59]. These previous investigations indicate that the same miRNAs exhibit multiple regulatory activities in plant responses to different abiotic stresses, which further supports the diverse functions of miRNAs in plants. Additionally, miRNAs are partially conserved in response to the same abiotic stress (Figure 1).

## 3. Stress-Responsive Long Non-Coding RNAs

Compared with small non-coding RNAs, lncRNAs are transcripts of at least 200 nt in length that possess no coding capacity and are involved in the regulation of various biological processes, including plant growth, development, and stress responses [8,60]. In *P. tomentosa* under N deficiency, the global characterization of lncRNAs revealed that 388 unique lncRNA candidates belonging to 380 gene loci were detected, and only 7 lncRNAs were found to belong to seven conserved non-coding RNA families, indicating that the majority of lncRNAs are species specific. This investigation also presented the regulatory relationship between lncRNAs and their potential target genes [61]. Investigations revealed 504 drought-responsive lincRNAs in *P. trichocarpa* [62]. Strand-specific RNA sequencing uncovered 204 high-temperature-responsive lncRNAs in *P*. *simonii*; these ncRNAs could regulate their target genes by acting as potential RNA scaffolds or through the RNA interference pathway. Furthermore, heterogeneous expression of targets from two heat-responsive lncRNAs promote photosynthetic protection and recovery, inhibit membrane peroxidation, and suppress DNA damage in *Arabidopsis* under heat stress [63]. In *Betula platyphylla* (birch), 30 lncRNAs (16 upregulated and 14 downregulated) were differentially expressed under cadmium (Cd) treatment. Moreover, nine lncRNAs were transiently overexpressed in birch in an exploration of their roles in Cd tolerance; it was suggested that lncRNAs can up- or down-regulate their target genes to improve Cd tolerance by transient over-expression, which increased our understanding of lncRNA-mediated Cd tolerance [64]. The over-expression of the lncRNA *Ptlinc*-*NAC72* confirmed that *Ptlinc*-*NAC72* can directly upregulate *PtNAC72*.*A*/*B* expression by recognizing the tandem elements (GAAAAA) in the *PtNAC72*.*A*/*B* 5′ untranslated region (UTR) under long-term salt stress, suggesting the important role of lncRNAs in *cis*- and *trans*-regulatory responses to salt stress in *P*. *trichocarpa* [65]. These findings highlight the potential contributions of lncRNAs in regulating the expression of plant genes that respond to abiotic stress. Additionally, it has been widely accepted that lncRNAs can form a regulatory network with other biological molecules, including target genes and miRNAs, thereby playing a pivotal role in modulating gene expression in *cis* or *trans* by participating in crucial pathways.

## 4. Stress-Responsive Circular RNAs

There have been few reports on circRNAs in plants, which are a newly discovered class of endogenous ncRNAs previously perceived as splicing errors, transcriptional noise, or artifacts [66]. CircRNAs are found in a wide range of organisms, and have been proposed to perform disparate functions. In general, circRNAs are thought to serve as miRNAs sponges, and they can also take part in protein or RNA transport [67]. CircRNA studies have been carried out in *Arabidopsis* [67], rice [68], maize [69], tomatoes [70], and soybeans [71].

In woody plants, the global profile has confirmed that circRNAs play important roles in wood formation for acclimation to low nitrogen stress in *P*. *canescens.* Networks of circRNAs–miRNAs–mRNAs are involved in wood formation under low N stress. In the circRNA392/1732/1226–miR169b–NFYA10/1-A/A1-B network, the upregulation of circRNAs causes increased transcription factor NFYA via the modulation of miR169b members in the wood of low-N-treated poplars, probably resulting in reduced xylem width and cell layers of the xylem [72]. In *Pyrus betulifolia Bunge*, 899 circRNAs were detected, among which 33 (23 upregulated, 10 downregulated) were shown to be dehydration-responsive using deep sequencing. Additionally, 309 circRNAs were predicted to act as sponges for 180 miRNAs, suggesting a circRNA-miRNA co-expression network between the differentially expressed circRNAs and their miRNA binding sites [73]. Under heat stress, *PtoXBAT32.5* expression was induced with upregulation of Circ0003418, indicating that Circ0003418 is a negative regulator of *P*. *tomentosa* heat tolerance via the ubiquitin-mediated protein modification pathway [74]. The interaction and molecular mechanisms of such regulation could provide critical insights into the understanding of gene regulation in plants under stress, which should accelerate the mining of major regulatory genes in plant stress resistance and applications of the molecular breeding of plants, especially for forest trees.

## 5. The lncRNA/circRNA–miRNA–mRNA Regulatory Network Involved in Abiotic Stress in Forest Trees

**Figure 2:**
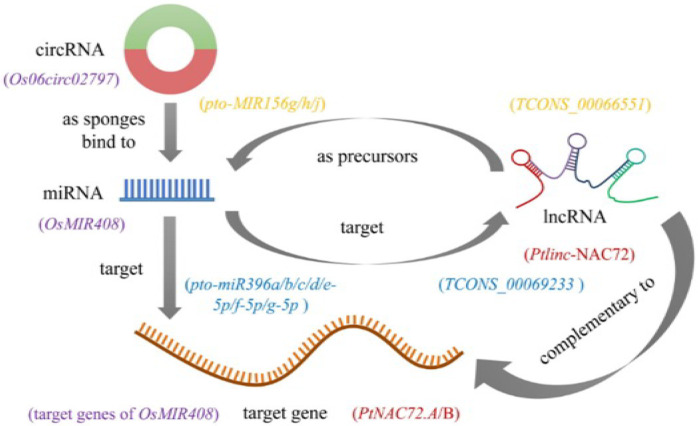
A model of intersections and feedback regulation between stress-responsive miRNAs, lncRNAs, circRNAs, and target genes [61,65,75]. Note: purple words are an example for a circRNA–miRNA–mRNA regulatory network; red, yellow and blue words are examples for lncRNA–miRNA–mRNA regulatory networks.

Each type of ncRNA has a unique method of performing biological functions. miRNAs have been studied for two decades, and their critical functions in diverse biological processes are better known than those of other ncRNAs. Many miRNA target genes have been identified and demonstrated to be vital for plant development and stress resistance. LncRNAs have attracted extensive attention in recent years, especially with the advancement of sequencing technologies and bioinformatics methods. Many lncRNA transcripts are precursors of known or novel miRNAs. For example, in *P. tomentosa*, 9 lincRNAs were detected as precursors of 11 known miRNAs, and 5 lncRNAs were identified as precursors of 14 novel miRNAs [61]. Additionally, the lncRNA TCONS_00066551 is aligned with the miRNA precursors pto-MIR156g/h/j at 114-216 nt. LncRNA transcripts may also be targets of miRNAs; 4 lncRNAs in *Populus* were predicted to be targeted by 29 miRNAs belonging to five families. Specifically, seven members of pto-miR396 (pto-miR396a/b/c/d/e-5p/f-5p/g-5p) were identified to target TCONS_00069233 (upstream of Potri.018G126700.1) [61]. These findings not only suggest the potential roles of lncRNAs involved in regulating plant stress responses, but provide new insights into the lncRNA–miRNA–mRNA regulatory network in plants. Additionally, expressed lncRNAs execute their functions in regulating stress-responsive gene expression either in a *cis*- or *trans*-acting manner, through binding to DNA/RNA in sequence complementarity. For instance, *Ptlinc*-*NAC72* targets *PtNAC72*.*A*/*B* in *P*. *trichocarpa* [65]. For circRNAs, previous investigation has found that *Os06circ02797* can be acquired by the mutant multiplexed CRISPR–Cas9 strategy. Furthermore, molecular and computational analyses indicated a circRNA–miRNA–mRNA regulatory network, where *Os06circ02797* functioned as a sponge for *OsMIR408*, which probably helped fine-tune the expression of *OsMIR408* target genes [75]. It should be noted that, so far, the identification of most lncRNAs and circRNAs has mainly been based on bioinformatics predictions. More detailed molecular and genetic analyses are urgently required to elucidate the regulatory modes of the biological functions of ncRNAs. Compared with lncRNAs and circRNAs, miRNAs might be at the hub of regulatory networks, but the mysterious nature of ncRNAs opens new possibilities for sophisticated regulatory mechanisms awaiting further exploration (Figure 2).

## 6. Non-Coding RNAs Involved in Epigenetic Modulation of Gene Expression under Abiotic Stress

Epigenetic modifications are important in the regulation of abiotic stress responses [76]. Both DNA methylation and post-transcriptional RNA modifications are plant epigenetic regulators [77]. In *P. trichocarpa* from different sites, differentially methylated miRNAs, together with their target genes, indicated not only a site-dependent, but also a Pi-dependent, expression profile, which implied epigenetic regulation might occur by RNA interference by differentially methylated miRNAs [78]. In *P. simonii*, 16 miRNAs and 17 lncRNAs were found in stress-specific differentially methylated regions under diverse stresses, including heat, cold, osmotic, and salt stress. Among methylated miRNA genes, only the expression of *MIRNA6445a* showed long-term stability. Furthermore, expression patterns of ncRNAs and their putative target genes differed under abiotic stress, suggesting the key role of ncRNAs in plant responses to abiotic stress through epigenetic modification [79]. Whole-genome analysis of gene expression and methylation patterns identified 1066 differentially methylated sites in *P. simonii’s* response to low temperature stress, and seven responsive miRNAs were identified by BLAST against miRBase. Furthermore, qRT-PCR revealed that miRNA gene methylation patterns may influence their expression. The network of DNA methylation, miRNAs, target genes, the products of target genes, and the metabolic factors that they affect suggest that DNA methylation probably regulates the expression of miRNAs, thus affecting the expression of miRNAs target genes, likely through the gene-silencing function of miRNAs, to maintain cell survival under abiotic stress conditions [80].

## 7. Discussion and Perspectives

**Figure 3:**
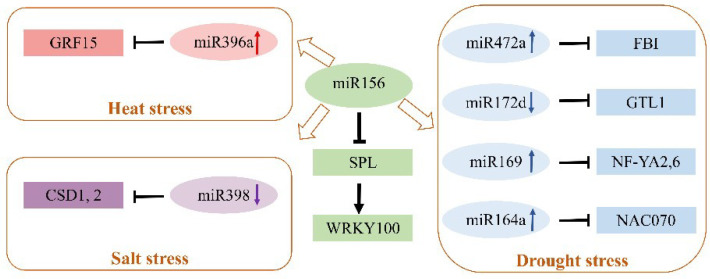
The regulatory network of miR156 for other miRNAs in forest trees in response to abiotic stress [24,25,26,35,52,85,86,87]. miR156 downregulates *SPL*, and the miR156/SPL module regulates salt stress tolerance in apple by activating *WERKY100* expression. Overexpression of miR156 upregulates miR472a, miR169, miR164a, and miR396a, while it downregulates miR172d and miR398 in poplar. miR396a mediates the response of heat stress via targeting *GRF15* in poplar. miR398 mediates the response of salt stress via targeting copper/zinc superoxide dismutase (*CDS1*,*2*) in grapevines. miR169o plays a positive role in regulating drought tolerance and growth by targeting the subunit A of the Nuclear Factor Y (*NF*-*YA6*) gene in poplar. miR172d regulates the drought stress response via targeting the GT-2-like 1 (*GTL1*) trihelix transcription factor in poplar. miR472a mediates the drought stress response via targeting F-box 1 (*FB1*) in poplar. miR164a mediates the drought stress response via targeting *NAC* (NAM, ATAF, and CUC) in poplar. Note: the brown arrows show miR156 indirectly mediates abiotic stress by regulating other miRNAs in forest trees. The black arrows show positive regulation and the black blocked arrows show negative regulation. The upward arrows behind miRNAs represent upregulated expression of miRNAs, the downward arrows behind miRNAs represent downregulated expression of miRNAs, and the rectangular box shows the target genes of miRNAs.

Intercellular and systemic trafficking of miRNAs was observed in plants. miRNAs can be transported to distant tissues through the phloem. In addition, small RNAs exchange information between species, which requires further investigation for other miRNAs or plants with this widely existing regulation [81]. Notably, one miRNA might have multiple target genes, and thus one miRNA might participate in several stress responses; one target gene could be regulated by several miRNAs, and thus multiple miRNAs might be involved in the same stress response in plants. It would be interesting to explore the intersection of stress responsive miRNAs and the regulatory relationship between miRNAs and their target genes in plants. During plant development and resistance to various abiotic stresses, miRNAs play crucial roles in regulating various target genes, which work as nodes and are organized into complex gene networks. miRNAs and target genes comprise a complicated regulatory network controlling plant resistance to multiple stimuli. For example, miR156, a conserved miRNA, plays crucial roles in various stresses, such as low Pi, drought, salinity, heat, cold, and heavy metal stress in plants [82,83,84]. Integrated miRNAomic and transcriptomic analysis has suggested that expression of miR472a, miR169, miR164a, and miR396a was upregulated, while expression of miR172d and miR398 was decreased, in transgenic poplar over-expressing miR156 [85]. Furthermore, miR472a, miR172d, miR169, miR164a and their target genes are involved in plant responses to drought stress; miR396a and its target gene are heat stress-responsive regulators; and miR398 with its target genes contributes to salt tolerance of woody plants [24,25,26,35,52,86]. Additionally, it has been revealed that the miR156/SPL module regulates salt stress tolerance by activating *MdWERKY100* expression in transgenic apple plants [87]. miR156 might indirectly affect the expression level of other miRNAs, revealing a regulatory network of miRNAs and their targets (Figure 3).

Until now, most miRNA studies utilized in silico analyses, and the results require further confirmation through experimental validation. The most frequently adopted method is genetic transformation investigation, involving increasing or suppressing the expression of miRNAs in plants and observing the resultant phenotypic changes. To date, several key techniques have been used to explore the regulatory functions of miRNAs and their targets: overexpressing miRNAs by transforming the precursor sequence into the plant, suppressing miRNA expression by transformation with short tandem target mimics (STTM), RNA interference (RNAi), and the clustered regulatory interspaced short palindromic repeats (CRISPR)/CRISPR-associated protein (Cas) system. Related work has also been carried out successfully in forest trees such as *Populus*. For example, knockdown of miR393 promoted growth and biomass production in poplar, which was verified in STTM393 transgenic poplar lines [88]. RNAi suppression of DNA methylation affected the drought stress response and genome integrity in transgenic poplar [89]. Additionally, expression of artificial microRNAs (amiRNAs) in plants using virus- and non-virus-based expression vectors achieved RNAi effects on specific transcripts [90]. Efficient CRISPR/Cas9-mediated genome editing was achieved in an interspecific hybrid poplar with a highly heterozygous genome [91]. Genome editing without introduction of exogenous genes could be the most promising technique for breeding forest trees with more abiotic stress tolerance. Molecular design breeding has been realized in forest trees; however, due to the difficulties of creating transgenic trees, small RNA functional verification still has a great deal of mining potential in forest trees. The comparison of miRNAs between *P*. *tomentosa* and other species showed that almost all the conserved miRNAs found in *P*. *tomentosa* were present in *P*. *trichocarpa*, *Arabidopsis*, rice, and maize, implying the conservation of miRNA among species [47]. Although the research of ncRNAs in forest trees lags behind that of crop species, the results for crops can be used as a reference for forest trees. Conversely, due to the long lifetime of forest trees, perennial trees might have evolved more coding genes, ncRNAs regulation, or epigenetic modification responses to abiotic stress compared with annual plants, which might be critical for resistant molecular breeding of crops.

## Supplementary Materials

The following supporting information can be downloaded at: https://www.mdpi.com/article/10.3390/ijms23126365/s1.

## References

[1] Yu Y., Zhang Y., Chen X., Chen Y. (2019). Plant Noncoding RNAs: Hidden players in development and stress responses. Annu. Rev. Cell Dev. Biol..

[2] Rogers K., Chen X. (2013). Biogenesis, turnover, and mode of action of plant microRNAs. Plant Cell.

[3] Wu L., Zhou H., Zhang Q., Zhang J., Ni F., Liu C., Qi Y. (2010). DNA methylation mediated by a microRNA pathway. Mol. Cell.

[4] Chellappan P., Xia J., Zhou X., Gao S., Zhang X., Coutino G., Vazquez F., Zhang W., Jin H. (2010). siRNAs from miRNA sites mediate DNA methylation of target genes. Nucleic Acids Res..

[5] Jones-Rhoades M.W., Bartel D.P., Bartel B. (2006). MicroRNAS and their regulatory roles in plants. Annu. Rev. Plant Biol..

[6] Liang C., Cai Q., Wang F., Li S., You C., Xu C., Gao L., Cao D., Lan T., Zhang B. (2022). *Arabidopsis* RBV is a conserved WD40 repeat protein that promotes microRNA biogenesis and ARGONAUTE1 loading. Nat. Commun..

[7] Wang K.C., Chang H.Y. (2011). Molecular mechanisms of long noncoding RNAs. Mol. Cell.

[8] Zhang Y., Tao Y., Liao Q. (2018). Long noncoding RNA: A crosslink in biological regulatory network. Brief. Bioinform..

[9] Xu H., Chen B., Zhao Y., Guo Y., Liu G., Li R., Zeisler-Diehl V.V., Chen Y., He X., Schreiber L. (2022). Non-Coding RNA analyses of seasonal cambium activity in *Populus tomentosa*. Cells.

[10] Zuo J., Wang Q., Zhu B., Luo Y., Gao L. (2016). Deciphering the roles of circRNAs on chilling injury in tomato. Biochem. Biophys. Res. Commun..

[11] Waititu J.K., Zhang C., Liu J., Wang H. (2020). Plant non-coding RNAs: Origin, biogenesis, mode of action and their roles in abiotic stress. Int. J. Mol. Sci..

[12] Oldroyd G.E.D., Leyser O. (2020). A plant’s diet, surviving in a variable nutrient environment. Science.

[13] Cheng X., He Q., Tang S., Wang H., Zhang X., Lv M., Liu H., Gao Q., Zhou Y., Wang Q. (2021). The miR172/IDS1 signaling module confers salt tolerance through maintaining ROS homeostasis in cereal crops. New Phytol..

[14] Gao Y., Feng B., Gao C., Zhang H., Wen F., Tao L., Fu G., Xiong J. (2022). The evolution and functional roles of miR408 and its targets in plants. Int. J. Mol. Sci..

[15] Bai Q., Wang X., Chen X., Shi G., Liu Z., Guo C., Xiao K. (2018). Wheat miRNA TaemiR408 acts as an essential mediator in plant tolerance to Pi deprivation and salt stress via modulating stress-associated physiological processes. Front. Plant Sci..

[16] Hang N., Shi T., Liu Y., Ye W., Taier G., Sun Y., Wang K., Zhang W. (2021). Overexpression of Os-microRNA408 enhances drought tolerance in perennial ryegrass. Physiol. Plant..

[17] Shuai P., Liang D., Zhang Z., Yin W., Xia X. (2013). Identification of drought-responsive and novel *Populus trichocarpa* microRNAs by high-throughput sequencing and their targets using degradome analysis. BMC Genom..

[18] Fang L., Wang Y. (2021). MicroRNAs in woody plants. Front. Plant Sci..

[19] Li B., Qin Y., Duan H., Yin W., Xia X. (2011). Genome-wide characterization of new and drought stress responsive microRNAs in *Populus euphratica*. J. Exp. Bot..

[20] Ren Y., Chen L., Zhang Y., Kang X., Zhang Z., Wang Y. (2012). Identification of novel and conserved *Populus tomentosa* microRNA as components of a response to water stress. Funct. Integr. Genom..

[21] Shuai P., Su Y., Liang D., Zhang Z., Xia X., Yin W. (2016). Identification of phasiRNAs and their drought- responsiveness in *Populus trichocarpa*. FEBS Lett..

[22] Deng M., Cao Y., Zhao Z., Yang L., Zhang Y., Dong Y., Fan G. (2017). Discovery of microRNAs and their target genes related to drought in *Paulownia* “Yuza 1” by high-throughput sequencing. Int. J. Genom..

[23] Cao X., Fan G., Cao L., Deng M., Zhao Z., Niu S., Wang Z., Wang Y. (2017). Drought stress-induced changes of microRNAs in diploid and autotetraploid *Paulownia tomentosa*. Genes Genom..

[24] Su Y. (2018). Functional Studies on Poplar miR472a and miR6445 in *Cytspora Chrysosperma* Infection and Drought Stress Response. Ph.D. Thesis.

[25] Jiao Z., Lian C., Han S., Huang M., Shen C., Li Q., Niu M., Yu X., Yin W., Xia X. (2021). PtmiR169o plays a positive role in regulating drought tolerance and growth by targeting the *PtNF*-*YA6* gene in poplar. Environ. Exp. Bot..

[26] Liu Q., Wang Z., Yu S., Li W., Zhang M., Yang J., Li D., Yang J., Li C. (2021). Pu-miR172d regulates stomatal density and water-use efficiency via targeting *PuGTL1* in poplar. J. Exp. Bot..

[27] Megha S., Basu U., Kav N.N.V. (2018). Regulation of low temperature stress in plants by microRNAs. Plant Cell Environ..

[28] Lu S., Sun Y., Chiang V.L. (2008). Stress-responsive microRNAs in Populus. Plant J..

[29] Chen L., Zhang Y., Ren Y., Xu J., Zhang Z., Wang Y. (2012). Genome-wide identification of cold-responsive and new microRNAs in *Populus tomentosa* by high-throughput sequencing. Biochem. Biophys. Res. Commun..

[30] Niu J., Wang J., Hu H., Chen Y., An J., Cai J., Sun R., Sheng Z., Liu X., Lin S. (2016). Cross-talk between freezing response and signaling for regulatory transcriptions of MIR475b and its targets by miR475b promoter in *Populus suaveolens*. Sci. Rep..

[31] Bao H., Chen M., Chen H., Du L., Wang Y. (2019). Transcriptome-wide identification of miRNA targets and a TAS3-homologous gene in Populus by degradome sequencing. Genes Genom..

[32] Ding Q., Zeng J., He X. (2014). Deep sequencing on a genome-wide scale reveals diverse stage-specific microRNAs in cambium during dormancy-release induced by chilling in poplar. BMC Plant Biol..

[33] Zhang Y., Wang Y., Gao X., Liu C., Gai S. (2018). Identification and characterization of microRNAs in tree peony during chilling induced dormancy release by high-throughput sequencing. Sci. Rep..

[34] Chen B., Xu H., Guo Y., Grünhofer P., Schreiber L., Lin J., Li R. (2021). Transcriptomic and epigenomic remodeling occurs during vascular cambium periodicity in *Populus tomentosa*. Hortic. Res..

[35] Zhao Y., Xie J., Wang S., Xu W., Chen S., Song X., Lu M., El-Kassaby Y.A., Zhang D. (2021). Synonymous mutation in growth regulating factor 15 of miR396a target sites enhances photosynthetic efficiency and heat tolerance in poplar. J. Exp. Bot..

[36] Zhao J., He Q., Chen G., Wang L., Jin B. (2016). Regulation of non-coding RNAs in heat stress responses of plants. Front. Plant Sci..

[37] Chen L., Ren Y., Zhang Y., Xu J., Sun F., Zhang Z., Wang Y. (2012). Genome-wide identification and expression analysis of heat-responsive and novel microRNAs in *Populus tomentosa*. Gene.

[38] Kumar V., Khare T., Shriram V., Wani S.H. (2018). Plant small RNAs: The essential epigenetic regulators of gene expression for salt-stress responses and tolerance. Plant Cell Rep..

[39] Li B., Duan H., Li J., Deng X.W., Yin W., Xia X. (2013). Global identification of miRNAs and targets in *Populus euphratica* under salt stress. Plant Mol. Biol..

[40] Si J., Zhou T., Bo W., Xu F., Wu R. (2014). Genome-wide analysis of salt-responsive and novel microRNAs in *Populus euphratica* by deep sequencing. BMC Genet..

[41] Ren Y., Chen L., Zhang Y., Kang X., Zhang Z., Wang Y. (2013). Identification and characterization of salt-responsive microRNAs in *Populus tomentosa* by high-throughput sequencing. Biochimie.

[42] Zhou J., Liu M., Jiang J., Qiao G., Lin S., Li H., Xie L., Zhuo R. (2012). Expression profile of miRNAs in *Populus cathayana* L. and *Salix matsudana* Koidz under salt stress. Mol. Biol. Rep..

[43] He F., Xu C., Fu X., Shen Y., Guo L., Leng M., Luo K. (2018). The MicroRNA390/trans-acting short interfering RNA3 module mediates lateral root growth under salt stress via the auxin pathway. Plant Physiol..

[44] Qiu T., Du K., Jing Y., Zeng Q., Liu Z., Li Y., Ren Y., Yang J., Kang X. (2021). Integrated transcriptome and miRNA sequencing approaches provide insights into salt tolerance in allotriploid *Populus cathayana*. Planta.

[45] Chien P., Chiang C., Wang Z., Chiou T. (2017). MicroRNA-mediated signaling and regulation of nutrient transport and utilization. Curr. Opin. Plant Biol..

[46] Chiou T. (2007). The role of microRNAs in sensing nutrient stress. Plant Cell Environ..

[47] Ren Y., Sun F., Hou J., Chen L., Zhang Y., Kang X., Wang Y. (2015). Differential profiling analysis of miRNAs reveals a regulatory role in low N stress response of Populus. Funct. Integr. Genomic..

[48] Bao H., Chen H., Chen M., Xu H., Huo X., Xu Q., Wang Y. (2019). Transcriptome-wide identification and characterization of microRNAs responsive to phosphate starvation in *Populus tomentosa*. Funct. Integr. Genomic..

[49] Zhang J., Lin Y., Wu F., Zhang Y., Cheng L., Huang M., Tong Z. (2021). Profiling of microRNAs and their targets in roots and shoots reveals a potential miRNA-mediated interaction network in response to phosphate deficiency in the forestry tree *Betula luminifera*. Front. Genet..

[50] Van Dongen J.T., Licausi F. (2015). Oxygen sensing and signaling. Annu. Rev. Plant Biol..

[51] Sunkar R., Chinnusamy V., Zhu J., Zhu J. (2007). Small RNAs as big players in plant abiotic stress responses and nutrient deprivation. Trends Plant Sci..

[52] Leng X., Wang P., Zhu X., Li X., Zheng T., Shangguan L., Fang J. (2017). Ectopic expression of *CSD1* and *CSD2* targeting genes of miR398 in grapevine is associated with oxidative stress tolerance. Funct. Integr. Genom..

[53] Fukao T., Bailey-Serres J. (2004). Plant responses to hypoxia-is survival a balancing act?. Trends Plant Sci..

[54] Kaling M., Kanawati B., Ghirardo A., Albert A., Winkler J.B., Heller W., Barta C., Loreto F., Schmitt-Kopplin P., Schnitzler J.P. (2015). UV-B mediated metabolic rearrangements in poplar revealed by non-targeted metabolomics. Plant Cell Environ..

[55] Jenkins G.I. (2009). Signal transduction in responses to UV-B radiation. Annu. Rev. Plant Biol..

[56] Jia X., Ren L., Chen Q., Li R., Tang G. (2009). UV-B-responsive microRNAs in *Populus tremula*. J. Plant Physiol..

[57] Sunitha S., Loyola R., Alcalde J.A., Arce-Johnson P., Matus J.T., Rock C.D. (2019). The role of UV-B light on small RNA activity during grapevine berry development. G3 (Bethesda).

[58] Niu S., Wang Y., Zhao Z., Deng M., Cao L., Yang L., Fan G. (2016). Transcriptome and degradome of microRNAs and their targets in response to drought stress in the plants of a diploid and its autotetraploid *Paulownia australis*. PLoS ONE.

[59] Chen M., Bao H., Wu Q., Wang Y. (2015). Transcriptome-wide identification of miRNA targets under nitrogen deficiency in *Populus tomentosa* using degradome sequencing. Int. J. Mol. Sci..

[60] Quan M., Chen J., Zhang D. (2015). Exploring the secrets of long noncoding RNAs. Int. J. Mol. Sci..

[61] Chen M., Wang C., Bao H., Chen H., Wang Y. (2016). Genome-wide identification and characterization of novel lncRNAs in Populus under nitrogen deficiency. Mol. Genet. Genom..

[62] Shuai P., Liang D., Tang S., Zhang Z., Ye C.Y., Su Y., Xia X., Yin W. (2014). Genome-wide identification and functional prediction of novel and drought-responsive lincRNAs in *Populus trichocarpa*. J. Exp. Bot..

[63] Song Y., Chen P., Liu P., Bu C., Zhang D. (2020). High-temperature-responsive poplar lncRNAs modulate target gene expression via RNA interference and act as RNA scaffolds to enhance heat tolerance. Int. J. Mol. Sci..

[64] Wen X., Ding Y., Tan Z., Wang J., Zhang D., Wang Y. (2020). Identification and characterization of cadmium stress-related lncRNAs from *Betula platyphylla*. Plant Sci..

[65] Ye X., Wang S., Zhao X., Gao N., Wang Y., Yang Y., Wu E., Jiang C., Cheng Y., Wu W. (2022). Role of lncRNAs in *cis*- and *trans*-regulatory responses to salt in *Populus trichocarpa*. Plant J..

[66] Meng X., Li X., Zhang P., Wang J., Zhou Y., Chen M. (2017). Circular RNA: An emerging key player in RNA world. Brief. Bioinform..

[67] Chen G., Cui J., Wang L., Zhu Y., Lu Z., Jin B. (2017). Genome-wide identification of circular RNAs in *Arabidopsis thaliana*. Front. Plant Sci..

[68] Lu T., Cui L., Zhou Y., Zhu C., Fan D., Gong H., Zhao Q., Zhou C., Zhao Y., Lu D. (2015). Transcriptome-wide investigation of circular RNAs in rice. RNA.

[69] Chen L., Zhang P., Fan Y., Lu Q., Li Q., Yan J., Muehlbauer G.J., Schnable P.S., Dai M., Li L. (2018). Circular RNAs mediated by transposons are associated with transcriptomic and phenotypic variation in maize. New Phytol..

[70] Tan J., Zhou Z., Niu Y., Sun X., Deng Z. (2017). Identification and functional characterization of tomato circRNAs derived from genes involved in fruit pigment accumulation. Sci. Rep..

[71] Zhao W., Cheng Y., Zhang C., You Q., Shen X., Guo W., Jiao Y. (2017). Genome-wide identification and characterization of circular RNAs by high throughput sequencing in soybean. Sci. Rep..

[72] Liu H., Yu W., Wu J., Li Z., Li H., Zhou J., Hu J., Lu Y. (2020). Identification and characterization of circular RNAs during wood formation of poplars in acclimation to low nitrogen availability. Planta.

[73] Wang J., Lin J., Wang H., Li X., Yang Q., Li H., Chang Y. (2018). Identification and characterization of circRNAs in *Pyrus betulifolia* Bunge under drought stress. PLoS ONE.

[74] Song Y., Bu C., Chen P., Liu P., Zhang D. (2021). Miniature inverted repeat transposable elements *cis*-regulate circular RNA expression and promote ethylene biosynthesis, reducing heat tolerance in *Populus tomentosa*. J. Exp. Bot..

[75] Zhou J., Yuan M., Zhao Y., Quan Q., Yu D., Yang H., Tang X., Xin X., Cai G., Qian Q. (2021). Efficient deletion of multiple circle RNA loci by CRISPR-Cas9 reveals Os06circ02797 as a putative sponge for OsMIR408 in rice. Plant Biotechnol. J..

[76] Miryeganeh M. (2021). Plants’ epigenetic mechanisms and abiotic stress. Genes.

[77] Meyer K.D., Jaffrey S.R. (2017). Rethinking m^6^A readers, writers, and erasers. Annu. Rev. Cell Dev. Biol..

[78] Schönberger B., Chen X., Mager S., Ludewig U. (2016). Site-dependent differences in DNA methylation and their impact on plant establishment and phosphorus nutrition in *Populus trichocarpa*. PLoS ONE.

[79] Song Y., Ci D., Tian M., Zhang D. (2016). Stable methylation of a non-coding RNA gene regulates gene expression in response to abiotic stress in *Populus simonii*. J. Exp. Bot..

[80] Ci D., Song Y., Tian M., Zhang D. (2015). Methylation of miRNA genes in the response to temperature stress in *Populus simonii*. Front. Plant Sci..

[81] Chen X., Rechavi O. (2021). Plant and animal small RNA communications between cells and organisms. Nat. Rev. Mol. Cell Biol..

[82] Lei K.J., Lin Y.M., Ren J., Bai L., Miao Y.C., An G.Y., Song C.P. (2016). Modulation of the phosphate-deficient responses by microRNA156 and its targeted SQUAMOSA PROMOTER BINDING PROTEIN-LIKE 3 in *Arabidopsis*. Plant Cell Physiol..

[83] Jerome J.J., Ali A., Wang W.M., Thiruvengadam M. (2020). Characterizing the role of the miR156-SPL network in plant development and stress response. Plants.

[84] Zhang L., Ding H., Jiang H., Wang H., Chen K., Duan J., Feng S., Wu G. (2020). Regulation of cadmium tolerance and accumulation by miR156 in *Arabidopsis*. Chemosphere.

[85] Wang Y., Liu W., Wang X., Yang R., Wu Z., Wang H., Wang L., Hu Z., Guo S., Zhang H. (2020). MiR156 regulates anthocyanin biosynthesis through SPL targets and other microRNAs in poplar. Hortic. Res..

[86] Lu X., Dun H., Lian C., Zhang X., Yin W., Xia X. (2017). The role of peu-miR164 and its target *PeNAC* genes in response to abiotic stress in *Populus euphratica*. Plant Physiol. Bioch..

[87] Ma Y., Xue H., Zhang F., Jiang Q., Yang S., Yue P., Wang F., Zhang Y., Li L., He P. (2021). The miR156/SPL module regulates apple salt stress tolerance by activating *MdWRKY100* expression. Plant Biotechnol. J..

[88] Chu L., He X., Shu W., Wang L., Tang F. (2021). Knockdown of miR393 promotes the growth and biomass production in poplar. Front. Plant Sci..

[89] Sow M.D., Le Gac A.L., Fichot R., Lanciano S., Delaunay A., Le Jan I., Lesage-Descauses M.C., Citerne S., Caius J., Brunaud V. (2021). RNAi suppression of DNA methylation affects the drought stress response and genome integrity in transgenic poplar. New Phytol..

[90] Kuo Y., Falk B.W. (2022). Artificial microRNA guide strand selection from duplexes with no mismatches shows a purine-rich preference for virus-and non-virus-based expression vectors in plants. Plant Biotechnol. J..

[91] Wang J., Wu H., Chen Y., Yin T. (2020). Efficient CRISPR/Cas9-mediated gene editing in an interspecific hybrid poplar with a highly heterozygous genome. Front. Plant Sci..

